# Performance and reproducibility of ^13^C and ^15^N hyperpolarization using a cryogen-free DNP polarizer

**DOI:** 10.1038/s41598-022-15380-7

**Published:** 2022-07-08

**Authors:** Arianna Ferrari, Josh Peters, Mariia Anikeeva, Andrey Pravdivtsev, Frowin Ellermann, Kolja Them, Olga Will, Eva Peschke, Hikari Yoshihara, Olav Jansen, Jan-Bernd Hövener

**Affiliations:** 1 Section Biomedical Imaging, MOIN CC, Department of Radiology and Neuroradiology, University Medical Center Schleswig-Holstein, Kiel University, Kiel, Germany; 2 Laboratory for Functional and Metabolic Imaging, Institute of Physics, EPFL (École polytechnique fédérale de Lausanne), Lausanne, Switzerland; 3 Department of Radiology and Neuroradiology, University Medical Center Schleswig-Holstein, Kiel University, Kiel, Germany

**Keywords:** Chemistry, Physics

## Abstract

The setup, operational procedures and performance of a cryogen-free device for producing hyperpolarized contrast agents using dissolution dynamic nuclear polarization (dDNP) in a preclinical imaging center is described. The polarization was optimized using the solid-state, DNP-enhanced NMR signal to calibrate the sample position, microwave and NMR frequency and power and flip angle. The polarization of a standard formulation to yield ~ 4 mL, 60 mM 1-^13^C-pyruvic acid in an aqueous solution was quantified in five experiments to P(^13^C) = (38 ± 6) % (19 ± 1) s after dissolution. The mono-exponential time constant of the build-up of the solid-state polarization was quantified to (1032 ± 22) s. We achieved a duty cycle of 1.5 h that includes sample loading, monitoring the polarization build-up, dissolution and preparation for the next run. After injection of the contrast agent in vivo, pyruvate, pyruvate hydrate, lactate, and alanine were observed, by measuring metabolite maps. Based on this work sequence, hyperpolarized ^15^N urea was obtained (P(^15^N) = (5.6 ± 0.8) % (30 ± 3) s after dissolution).

## Introduction

Magnetic Resonance Imaging (MRI) has revolutionized modern diagnostics by providing high resolution anatomical and functional imaging in 3D without ionizing radiation^1,2^. Many of the biochemical processes in vivo, however, still elapse our best efforts, and accessing these remains a prime objective of much research.

Here, hyperpolarized contrast agents hold great promise as they provide a unique window into metabolism, non-invasively and in vivo. By boosting the signal of selected, often endogenous molecules, their fate can be followed—for a limited time—with high spatial and chemical resolution. These properties have allowed the identification of cancerous tissue before a tumor was apparent, and has helped monitoring treatment response.

Dissolution dynamic nuclear polarization (dDNP)^3^ is the most established technique for hyperpolarizing (HP) biomolecules for in vivo imaging, and it shares the applicability to humans^4,5^ only with hyperpolarized Xenon^6^. Other HP techniques include brute-force^7^, parahydrogen-induced polarization^8^, chemically induced dynamic nuclear polarization^9^ and, for noble gases, spin-exchange optical pumping^10,11^.

dDNP has allowed to polarize biomolecules to more than 50% in about 1 h^12,13^. The nuclear polarization of the target is achieved by polarizing electronic spin first, using low temperatures (≈ 1.4 K) and high magnetic fields (≈ 6.7 T). Then, the electron polarization is transferred to nuclear polarization using the interactions between the electronic and nuclear spin under the action of electromagnetic waves, transmitted at a frequency corresponding to the difference in Larmor frequency of the two electron spins involved^14^. The unpaired electron spins are added in the form of stable radicals: TEMPO^15^, TEMPOL, or trityl radicals^16,17^ or induced by UV radiation^18^. In addition, there are other types of sample formulations, for instance HYPOP^19^.

When the desired level of nuclear spin polarization is achieved, the frozen sample is rapidly melted, dissolved and ejected from the polarizer by pressurized overheated water, such that an injectable contrast agent results.

Overall, dDNP is a complex process combining nuclear magnetic resonance (NMR) electron spin resonance (ESR), radical chemistry, high magnetic fields, fast dissolution, and cryogenictemperatures. Making this process available for biomedical research routinely is not straight forward. Over the last decades, several experimental implementations of dDNP were presented such as a cryogen-consumption-free DNP 9.4 T polarizer^20^, a 129-GHz dynamic nuclear polarizer in an ultra-wide bore superconducting magnet^21^, a Dynamic Nuclear Polarization Polarizer for sterile Use Intent^22^ and a multisample 7 T dynamic nuclear polarization polarizer for preclinical hyperpolarized MR^23^. Moreover, a number of dDNP polarizer were commercialized: HyperSense by Oxford Instruments^3^, SpinLab by GE and SpinAligner by Polarize^24^.

Here, we present our first experiences with the latest addition to the family, a cryogen-free dissolution polarizer for preclinical applications (SpinAligner, Polarize, Denmark)^24^. We report on the installation of the device and the routine for ^13^C and ^15^N hyperpolarization. By implementing routine operational procedures, high and reproducible polarization was achieved.

## Methods

### DNP system

**Figure 1:**
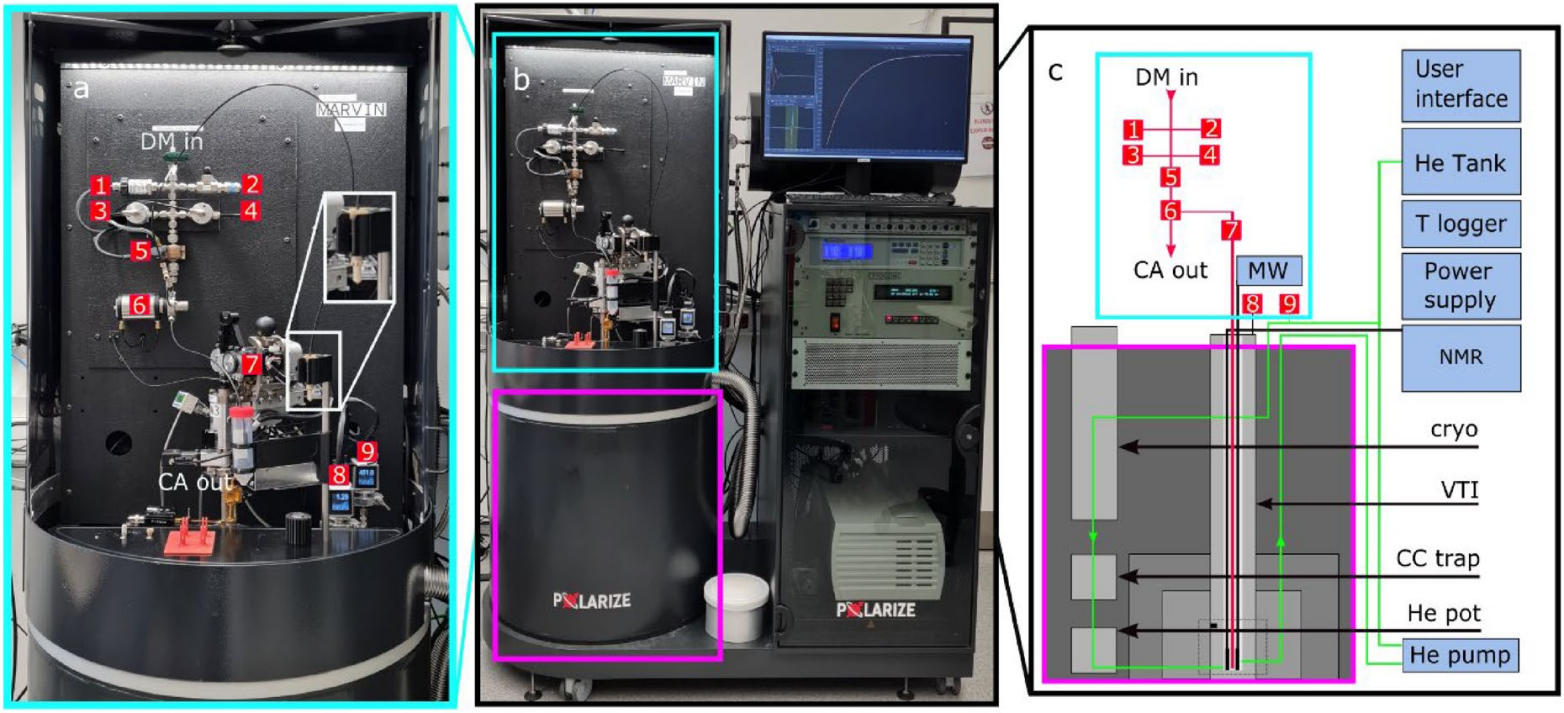
Photo (**a**,**b**) and diagram (**c**) of the polarizer used in this work. The polarizer consists of a dissolution module (**a**) mounted on the magnet (**b** section, magenta square) and a rack for auxiliary parts. The dissolution module (**a**) contains an inlet for the dissolution medium (DM), a heater for DM (5), a Swagelok manifold (1–4), a switch valve (6), the sample cup and an outlet. The magnet (**b** section, magenta square) is equipped with a variable temperature inset (VTI) which is cooled down to 1.4 K by evacuating a bath of liquid helium at the bottom of the VTI. The pressure of helium gas inside the VTI and in the inlet are shown by P1 (8) and P2 monitors respectively. The DNP probe inside the VTI consists of a tunnel for the sample cup, a waveguide to transmit the microwaves, and an NMR coil at the bottom. The sample is inserted into the VTI via an airlock (7) on top. The rack is used to house the pump circulating the Helium of the VTI, an NMR spectrometer, the magnet power supply, temperature controllers and the user interface. Figure **c** shows a schematic view of the polarizer naming all the main components.

**Figure 2:**
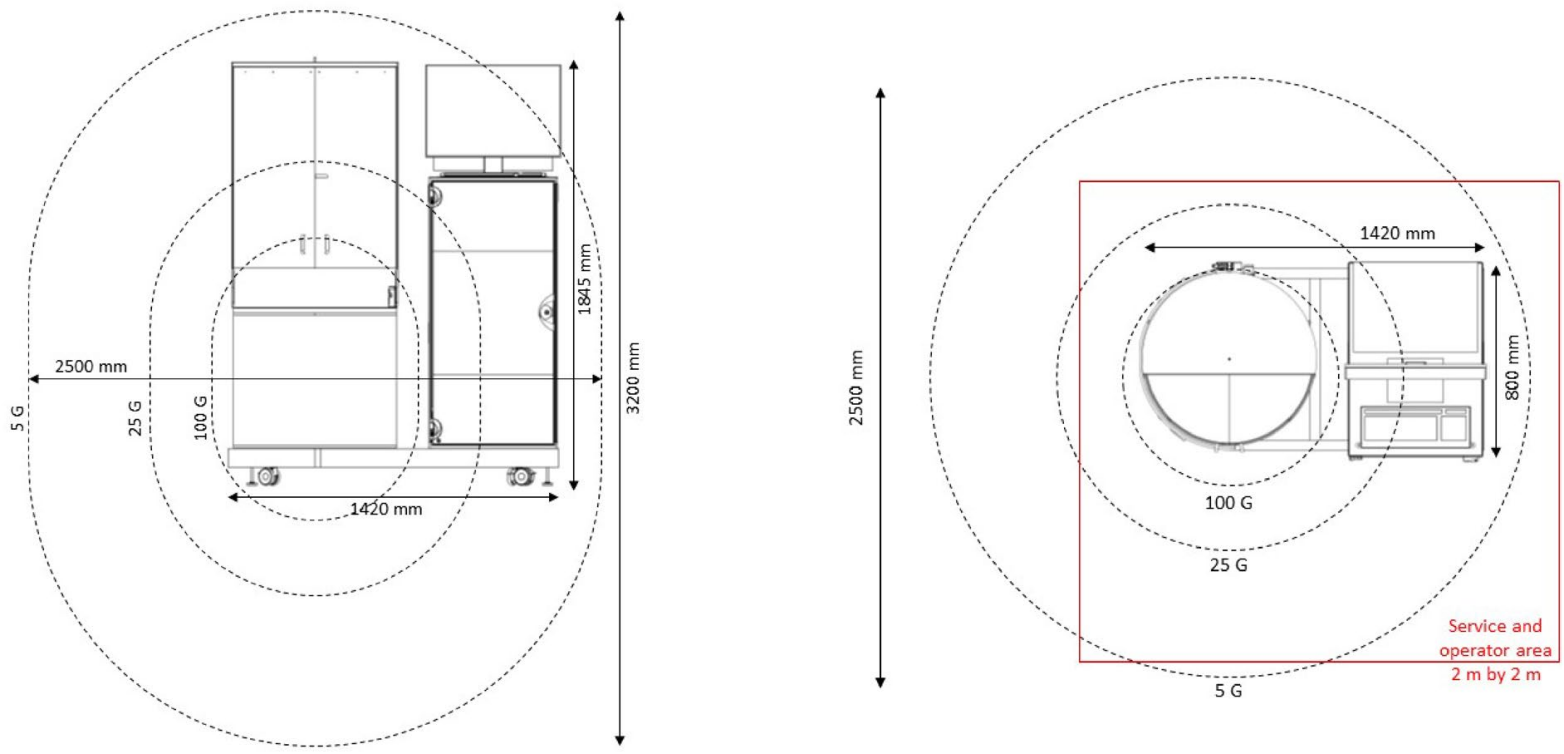
Drawings of the dDNP system indicating the dimension, stray magnetic field (dashed lines) and operator area (red line). (Drawings are reproduced from SpinAligner User Manual with permission).

The polarizer used in this work (SpinAligner, Polarize, Denmark) is similar to a setup described in 2019^24^, but features a different magnet (6.7–10.4 T) and dissolution module. The main components are a cryogen-free, superconducting magnet cooled by a closed-cycle helium cryostat, a variable temperature insert (VTI), a microwave source, an NMR spectrometer, a dissolution module, and control software (Figs. 1, 2). The superconducting magnet was driven by a 230 V/10 A power supply to reach max. 9.4 T (instead of 10.4 T as described in 2019^24^) and was used at ~ 6.7 T here. It is relevant to point out that the fluid path for dissolution is optimized for pre-clinical and in-vitro applications.

For hyperpolarization, c.a. 22 mg of a formulation containing the radical and concentrated contrast agent was filled into a sample cup (PEEK, maximum filling volume is 400 µL), lowered into the magnet and polarized. Once the desired polarization was reached, the sample was dissolved, expelled, and diluted by injecting a superheated dissolution medium into the cup. The cup was connected to the polarizer’s fluid system using a disposable O-ring seal, inserted into the VTI via an airlock (Fig. 1), and lowered into the magnet using a centrally controlled mechanism. In the VTI, temperatures below 1.5 K were reached by pumping on a liquid helium bath. The helium was pumped from a 50 L storage cylinder into the closed cooling circuit to replenish the helium bath after condensation at the magnets cryo-cooler. It returns back to the tank through a charcoal filter (inside the magnets housing A needle valve was used to control the helium supply to the VTI, and volumes were chosen never to exceed atmospheric pressure assuring safety. P1 and P2 monitors are showing He pressure inside the VTI and just outside of the He tank respectively. The temperature of the sample was estimated by a ceramic thermistor on the outside of the copper microwave cavity and the pressure inside the VTI, measured below the airlock.

An NMR spectrometer (Cameleon, Spinit, RS2D) was connected to an Alderman-Grant coil inside the VTI to acquire NMR signal in situ. The frequency and impedance of the NMR probe were adjusted using variable capacitors of an LC-circuit in an aluminum box outside of the bore. Adjusting or exchanging the circuit allowed acquiring ^1^H, ^13^C, ^15^N, ^63^Cu, or ^129^Xe signals.

A microwave source with a maximum continuous output power of 100 mW provided constant or frequency modulated^25^ irradiation of the sample in the VTI as set by the control software.

All procedures were controlled by a central software and digital-analog converters (Polarize; LabVIEW, National Instruments). Notably, data of more than 4 sensors was constantly monitored and stored in a compressed fashion.

The dDNP system was set up close to a 7 T MRI (30 cm bore, BioSpec 70/30, Bruker), two 1 T benchtop NMR spectrometers (Spinsolve Carbon and Nitrogen, Magritek) and a 9.4 T high-resolution wide-bore NMR spectrometer (9 cm bore, WB400, Avance NEO, 5 mm BBFO probe, Bruker). Once installed, a series of calibrations was performed (Table 2).

### Preparation of dDNP samples and dissolution medium

All the information about the standard sample are taken from the SpinAligner User Manual.

A general, step-by-step description of the preparation of contrast agent (CA)—radical concentrates is provided in Table 1; the procedure specifically for pyruvate is in Table 4 and can be adapted for other substances.

**Table 1:** General procedure to prepare contrast agent (CA) concentrates for DNP.

Step	Operation	Duration (min)	Comments
1	Take CA and trityl radical out of the freezer/fridge	5	Consider storing conditions and shelf life of agents
2	Wait until CA and radical are melted	20	Keep the vials in a warm place. Avoid the direct sunlight
3	Add desired amount of CA to the vial (e.g. 1.3 g)	2	e.g. Eppendorf vial
4	Add deionized water and glycerol in sufficient quantities (e.g. 0.5 g each)	2	Use scales to measure glycerol
			Check for the glassy matrix by dropping 5 µL of the solution in liquid nitrogen
5	Put desired amount of radical in a vial (e.g. ~ 50 mg)	2	
6	Mix CA and radical	10	Using spatula or ultrasonic mixer e.g
7	Divide in ca. 250 µL aliquots	5	Using pipet, Eppendorf vial
8	Put mixtures in freezer	1	e.g. − 20 °C
9	Warm up one vial, retrieve desired amount of concentrate, freeze remaining aliquot	15	

In general, a larger amount (e.g. 1.5 g) of CA-radical concentrate was prepared each time, split into smaller aliquots (e.g. 250 mg) and stored at − 22 °C. For a DNP experiment, one of these batches was warmed up, and the desired amount was retrieved (e.g. 22 mg).

All experiments were conducted using trityl radical (AH111501, molecular weight 1595 g/mol, POLARIZE) and one of the contrast agents described below.

#### Pyruvic acid ^13^C

About 1.5 mL Pyruvic acid radical concentrate was prepared and frozen in 250 µl aliquots at − 24 °C, containing 30 mM trityl radical and 14 M 1-^13^C-pyruvic acid (Table 2 1-^13^CPA, molecular weight 89.05 g/mol, Sigma-Aldrich, CAS: 99124-30-8). For each dDNP experiment, one aliquot was warmed up and the indicated amount of the concentrate was retrieved (typically 22 mg).

**Table 2:** Basic calibrations of NMR and DNP.

Step	Operation	Duration	Results and comments
1	**Calibration of NMR frequency using **^**63**^**Cu** Tune and match the NMR coil to estimated ν^63Cu^ Record ^63^Cu NMR signal and refine ν^63Cu^ Calculate *B*_0_, ν^13C^, ν^15N^, ν^e−^	2 min	*ν*^63Cu^ = 75.259 MHz ^63^Cu signal originates from the coil itself, no sample needed
2	**Fine calibration of NMR frequency ν**^**13C**^** or ν**^**15N**^** or other ν**^**X**^ Insert DNP sample Tune and match NMR coil to the desired nucleus Perform microwave frequency sweep Use hyperpolarized NMR signal to adjust RF transmitter		*ν*^13C^ = 71.492 MHz for pyruvate-radical concentrate *ν*^*15N*^ = 28.8225 MHz for urea-radical concentrate
3	**Optimize µW frequency** **ν**^**µW**^** and power** $p_{w}^{MW}$ Insert DNP sample (or leave it after step 2 inside) Tune and match NMR coil to desired nucleus Perform **µW** frequency and power sweep	ca. 60 min	
4	**Optimize sample position x**_**s**_** inside VTI** Insert DNP sample (or leave it after step 2 inside) Acquire NMR signal for different sample positions	ca. 30 min	Optimal position x_s_ found at 10 mm above bottom; closer to bottom assures colder sample temperature
5	**Calibration of the NMR flip angle** **α** Insert DNP sample and adjust coil Perform DNP until sufficient signal is obtained Apply train of low flip angle pulses Fit signals to obtain α for the given $p_{d}^{RF}$,$p_{a}^{RF}$		Several iterations may be needed to fulfill low flip angle condition. Depending on the sample position in the coil and VTI (Step 4)

Note that the results of some steps are dependable on another and may need to be repeated (e.g. No. 3–5).

#### Urea ^13^C, ^15^N

51 mg of trityl radical and 250 mg ^13^C,^15^N_2_-urea (molecular weight 60.05 g/mol, Sigma-Aldrich, CAS: 58069-83-3) were solved in 500 mg of deionized water and 500 mg of glycerol (G7893-500 mL, MW 92.09 g/mol, CAS: 56-81-5, Sigma-Aldrich). The resulting concentrate contained 35.5 mM of trityl radical and 4.62 M of urea and was stored at − 24 °C. For each DNP experiment, the indicated amount of the concentrate (typically 54 mg) was taken from the stock and transferred into the sample cup.

#### Urea ^13^ N

51 mg of trityl radical and 250 mg of [1,3-^15^N] urea (molecular weight 60.05 g/mol, Sigma-Aldrich, CAS: 2067-80-3) were solved in 500 mg of deionized water and 500 mg of glycerol (G7893-500 mL, MW 92.09 g/mol, CAS: 56-81-5, Sigma-Aldrich). The resulting concentrate contained 35.5 mM of trityl radical and 4.62 M of urea and was stored at − 24 °C. For each DNP experiment, the indicated amount of the concentrate (typically 54 mg) was taken from the stock and filled into the sample cup.

#### Dissolution medium

The dissolution medium was prepared by mixing 1.51 g of Trizma pre-set crystals (pH 7.6, average molecular weight 149.0 g/mol Sigma-Aldrich, T7943) to buffer the sample to obtain a final pH close to 7, 27 mg of ethylendiamintetracetic acid (EDTA, SERVA, CAS: 9002-07-7), 0.756 g of NaCl (Sigma-Aldrich) and 0.81 g of NaOH (Sigma-Aldrich, CAS: 1310-73-2) in 250 mL of deionized water and stored at − 20 °C. Typically, 3.9 mL were used for one experiment. For the urea experiments, only H_2_O with 0.27 mM EDTA was used instead.

### RF and µW frequency calibration

As indicated in the SpinAligner manual, after ramping up the magnet, the magnetic field strength was determined by detecting the solid-state ^63^Cu NMR signal of the NMR coil (*B*_0_ = 2π ν^63Cu^/*γ*^63Cu^) where ν^63Cu^ is the frequency of the ^63^Cu resonance and *γ*^63Cu^/2π = 11.319 MHz/T is its magnetogyric ratio. With *B*_0_, the frequencies of ^13^C, ^15^N, ^129^Xe and e^-^ were calculated correspondingly (*ν*_x_ = *B*_0_ *γ*_x_/2π) (see other *γ*_x_ values in SI). A finer calibration of the frequencies was performed using the NMR signal of the nuclei.

### The abbreviations used for NMR signal recording in Spinit

In the following, NA is number of averages per spectrum, NS is number of scans per spectrum, NX is number of excitations within the TR period, TR is the repetition time, and TX is the time between two consecutive excitations within one series of pulses. When we measured NMR spectra with DNP (Spinit, RS2D), we used often NS = 1 or 4, with TX < TR; the common parameters were TX = 217 µs and TR from 1 min to 1 h. When we measured NMR spectra with NMR spectrometers NS was 1 hence TX = TR.

### NMR flip angle calibration

The RF flip angle α was calibrated by using a train of low-flip angle excitations to a DNP-enhanced sample. 490 excitations were applied in 10 s (TR = 1 s, TX = 217 µs, NX = 49, NS = 1) resulting in an average number of excitations every second N = NX/TR = 49 s^−1^. Each 49th free induction decay (FID) was recorded and processed. Then we fitted a mono-exponential decay function to the data.

1 $$S^{obs} \left( t \right) = S_{0} \cdot e^{{ - \frac{t}{\tau }}}$$

(An extensive description of this approach is illustrated by Elliott et al.)^26^.

We will use the same function to fit the decay of polarization in liquid state to obtain apparent $T_{1}^{obs}$ (explained in more details below).

Assuming that the polarization didn’t relax significantly during the course of the experiment it allowed us to obtain the flip angle applied (Eq. 2, details in SI):

2 $${\upalpha } \cong arccos\left( {e^{{ - \frac{1}{{{\uptau } \cdot {\text{N}}}}}} } \right)$$

3 $$N = NX/TR$$

where S(t) is the signal acquired at time point t, *S*_0_ is the initial signal, ${\uptau }$ is the fitted constant, NX is total number of excitations per TR period, N is number of excitations per second.

Assuming linearity of the RF power amplifier this calibration was used to set the excitation angle for other durations $p_{d}^{RF}$ or power attenuation $p_{a}^{RF}$ (in dB) using the settings for the calibrated angle:

4 $$\alpha \left( {p_{d}^{RF} , p_{a}^{RF} } \right) = \alpha \left( {p_{d,ref}^{RF} , p_{a,ref}^{RF} } \right) \cdot \frac{{p_{d}^{RF} }}{{p_{d,ref}^{RF} }} \cdot 10^{{ - \frac{{p_{a}^{RF} - p_{a,ref}^{RF} }}{20}}}$$

### Microwave power and frequency calibration

The polarization transfer from electrons to nuclei was optimized by acquiring the DNP-enhanced ^13^C- or ^15^N-NMR signal as a function of microwave power (in W) $p_{w}^{{{\mathbf{\mu W}}}}$ (common increment of $p_{w}^{{{\mathbf{\mu W}}}}$ was 5 mW) and frequency ν^**µW**^ (common increment of ν^**µW**^ was 10 MHz). After 1–2 min of microwave irradiation, the solid state, ^13^C (or ^15^N)-NMR signal of DNP polarized CA-radical concentrate using a constant 2°–5° excitation pulse was measured for different settings of the **µW** frequency or power. One thousand pulses with the same flip angle were applied after each acquisition to saturate the remaining polarization.

### Optimization of sample position x_s_ in the VTI

A pyruvate-radical concentrate was polarized for 40 min and moved to different positions in the VTI, where ~ 2° ^13^C-spectra were acquired.

### Thermally polarized solid-state NMR and T_1_

To detect solid state ^13^C NMR signal in thermal equilibrium in the polarizer, 247.9 mg of pyruvate-radical concentrate (30 mM trityl-radical and 14 M 1-^13^C-PA) was prepared and inserted into the probe at ≈ 1.4 K. Every hour, several low-flip angle ^13^C NMR spectra were acquired to monitor the magnetization reaching the equilibrium ($\alpha$ ~ 0.32°, $p_{d}^{RF}$ = 2 us, $p_{a}^{RF}$ = 38 dB, NS = 256, TS = 217 us, and TR = 1 h). The data was exported and processed offline (baseline, phase correction, zero-filling to 4096 and integration, MestReNova). A mono-exponential recovery function (Eq. 4) was fitted to the data to obtain the apparent equilibrium signal $S_{inf}^{\alpha }$ and the apparent solid-state recovery time $T_{1}^{obs}$:

5 $$S^{obs} \left( t \right) = S_{inf}^{obs} \left( {1 - e^{{ - \frac{t}{{T_{1}^{obs} }}}} } \right).$$

Knowing excitation angle $\alpha$ and number of excitations per second $N$ we estimated real relaxation time and equilibrium signal (see SI for details) as

6 $$T_{1} = T_{1}^{obs} \left[ {1 + NT_{1}^{obs} \ln \left( {\cos \left( \alpha \right)} \right)} \right]^{ - 1} ,$$

7 $$S_{inf} = S_{inf}^{obs } \left[ {1 - NT_{1} \ln \left( {\cos \left( \alpha \right)} \right)} \right],$$

$S_{inf}$ is the equilibrium signal when complete equilibrium of the signal without RF excitations is reached and as before $N = NX/TR$ (Eq. 2).

### DNP-enhanced solid state nuclear polarization build-up

The build-up of the solid-state, DNP-enhanced polarization was monitored in situ using $\alpha \cong 0.7^\circ$ excitation with NS = 4 for ^13^C-DNP and about 3.5° for NS = 1 for ^15^N-DNP. The signals S(t) were automatically integrated and displayed on the polarizer along with an exponential recovery function fitted to the data. For a more detailed analysis, the spectra were processed offline (zero-filling, baseline correction, phase correction, integration; MestReNova). A mono-exponential recovery function (Eq. 5) was fitted to the data to obtain the build-up constant T^DNP^, taking the effect of the flip angle into account (Eq. 6).

### DNP-enhanced solid state NMR enhancement and polarization

The DNP signal enhancement *ε* of the solid sample in the polarizer was calculated using the signal intensities of the thermally and hyperpolarized spectra, taking into account the acquisition parameters (Eq. 8).

8 $$\varepsilon = \frac{{S^{{{\text{HP}}}} }}{{S^{{{\text{TP}}}} }} \cdot \frac{{NS_{{{\text{acq}}}}^{TP} }}{{NS_{{{\text{acq}}}}^{HP} }} \cdot \frac{{\sin \left( {\alpha_{TP} } \right)}}{{\sin \left( {\alpha_{HP} } \right)}} \cdot \frac{{RG_{TP} }}{{RG_{HP} }}$$

where *S* are the integrals over corresponding NMR signals, $NS_{{{\text{acq}}}}^{TP}$ and $NS_{{{\text{acq}}}}^{HP}$ are the number of scans for the thermally and hyperpolarized sample, and $\alpha_{HP}$ and $\alpha_{TP}$ are the excitation flip angles used to acquire the hyperpolarized and thermal spectra, respectively. $RG_{TP}$ and $RG_{HP}$ are values of linear receiver gains (to notice that T1 therefore S values correction are shown in SI).

The absolute polarization was calculated by multiplying the enhancement by the thermal polarization ($P^{{{\text{TP}}}}$, Eq. (9)): P^TP^(^13^C) (1 T, 295 K) = 0.8711 ppm, P^TP^(^13^C) (6.65 T, 1.4 K) = 1220 ppm, P^TP^(^13^C) (9.4 T, 295 K) = 8.187 ppm, (^15^N)P^TP^(^15^N) (9.4 T, 295 K) = 3.3 ppm.

9 $$P^{{{\text{TP}}}} = {\text{tanh}}\left( {\frac{{\hbar \gamma B_{0} }}{{2k_{{\text{B}}} T}}} \right)$$

10 $$P^{{{\text{HP}}}} = P^{{{\text{TP}}}} \cdot \varepsilon$$

where $\hbar$ is the reduced Planck constant, *ε* is the enhancement factor, *k*_B_ is the Boltzmann constant, *B*_0_ is the magnetic field and *T* is the temperature.

### Thermally polarized liquid-state NMR

Liquid state NMR was acquired either by a 1 T benchtop NMR (Spinsolve Carbon, Magritek) or a 9.4 T high-resolution NMR (WB400, Avance NEO, 5 mm BBFO probe, Bruker). The ^13^C or ^15^N signal intensities were quantified using automatic baseline and manual phase correction prior to numerical integration (integration region around the signal was ± 1 ppm at 9.4 T and, ± 2 ppm at 1 T, using TopSpin or MestReNova).

To accelerate the acquisition of ^13^C NMR of thermally polarized samples at 1 T, 4 vol% Gd-contrast agent was added ([Gd], 1 mmol/mL, Gadovist, Bayer). We used 3600 averages, flip angle 20°, TR = 2 s and RG = 31 (note that the same RG was used to acquire liquid state NMR spectra of the hyperpolarized solution). Estimated T_1_ was 50 ms.

To obtain thermally polarized ^13^C-signal at 9.4 T, we used a single scan with 90° flip angle, with RG = 101, 20 min after dissolution (RG = 0.25 for liquid state NMR spectra of the hyperpolarized solution).

For ^15^N NMR, 3 vol% [Gd] was added and 128 acquisitions after 90° flip angle were collected at 9.4 T using TR = 17 s. No thermal ^15^N signal was observed at 1 T in 100.000 averages and TR = 2 s.

### Liquid-state polarization decay

The hyperpolarized spectra were acquired after manual transfer to the respective device sequentially using fixed repetition time TR and a constant flip angle $\alpha_{HP}$.

To quantify lifetime of hyperpolarization $T_{1}^{HP}$, a mono-exponential decay function was fitted to the data yielding $T_{1}^{obs}$ (Eq. 1). $T_{1}^{obs}$ was corrected considering the polarization consumed by the repetitive RF excitations using Eq. (6) (Eq. 6, see details in SI).

### Liquid-state enhancement and polarization

The signal enhancement $\varepsilon$ and absolute polarization P was quantified with respect to the (averaged) signal from the thermally polarized samples using (Eqs. 5 and 7).

Note that all the experiments shown here were analyzed without any background subtractions.

### Animals

Two male FVB.TgN(Ela1KRAS.G12D)9EPS.CEABAC were bred at the Central Animal Facility of the University Hospital Schleswig–Holstein, Kiel, Germany. The animals were measured at ~ 9 months and had a weight of ~ 35 g. This study was conducted in compliance with the German Animal Protection Law. The animal protection committee of the local authorities (Ministry of Energy, Agriculture, the Environment, Nature and Digitalization Schleswig–Holstein (MELUND)) approved all experiments (V242-18779/2021(2-1/21)).

This study is reported in accordance with ARRIVE guidelines.

### HP-MRI in vivo

A mouse model for spontaneous pancreatic tumor was anaesthetized using intra peritoneal injection of 75 mg/kg ketamine and 0.5 mg/kg medetomidine. The anesthesia was diluted 1:5 with 0.9% NaCl yielding 155 µl solution per mouse. A tail vein catheter was used for injection of 200 µL HP 1-^13^C-PA solution. During the in vivo measurements the animals were heated via the bed and the vital parameters of the animals were monitored continuously. After the MRI measurements (~ 1 h), the animals were euthanized without awakening by cervical dislocation. Images were acquired on a 7 T, 30 cm MRI system (Biospec 70/30, Avance Neo, Bruker, Germany), equipped with a cylindrical, dual-tune ^1^H-/^13^C-volume transmit coil (72 mm diameter and 100 mm length and flexible ^13^C-surface receive coil (20 mm diameter, RAPID biomed, Germany)).

A T_2_-weighted ^1^H 2D RARE MRI was acquired for anatomical reference (acquisition time = 76.5 s, TR = 0.75 s, TE = 13 ms, 256 × 256 matrix size and field of view FOV = 33 mm × 33 mm, 9 slices with slice thickness = 4.26 mm, flip angle 90°/180°).

Free-induction decay 2D-chemical shift image (CSI) was acquired every 5 s for eight times in a row (acquisition time = 5 s, TR = 44.62 ms, TE = 0.489 ms, 11 × 11 matrix size, FOV = 33 mm × 33 mm, single slice, slice thickness = 4.26 mm, flip angle 5°)^27–29^.

^13^C-CSI images were processed using a script developed by Franz Schilling’s group (Matlab).

## Results

### DNP installation and initial calibration

#### Installation

The polarizer is mobile, mounted on wheels, (Fig. 1) and was placed at a distance of 3 m from the 7 T preclinical MRI, 2 m from two 1 T benchtop NMR spectrometers and 5 m from the 9.4 T NMR. The setup was provided with a 1-phase power outlet (10 A), dried and pressurized air (Atlas Copco 8F1 type air compressor), and Helium gas from a 50 L bottle (5.0 purity, Air Liquide). The helium compressor for cooling the magnet was installed at a distance of 9 m using a 20 m helium line. The He compressor requires cooling water and three phase electrical power (F-70H, Sumitomo).

The magnet was evacuated (HiCube 80 Classic vacuum pump, Pfeiffer Vacuum) before the closed-cycle He cooler was turned on (recommended pressure < 1 × 10^−4^ mbar). When a temperature of 1.5 K was reached (calculated from the pressure of the VTI outlet), the target field of 6.7 T was set in the polarizer user interface, and the magnet was ramped up in ~ 30 min to I = 72.811 A (using a factory calibration). The stabilizing period took around 20 min.

#### Calibration of RF frequency

The ^63^Cu-NMR signal of the NMR coil in the VTI was detected at ν^63Cu^ = 75.259 MHz using 4 averages and flip angle of 32° ($p_{a}^{RF}$ =  − 2 dB, $p_{d}^{RF}$ = 2 µs, see SI, Sect. 4.1.) resulting in *B*_0_ = 6.6489 T, ^13^C-frequency *ν*^13C^ = 71.197 MHz and electron Larmor frequency *ν*^e−^ = 186.397 GHz. The full width at half maximum (FWHM) of the ^63^Cu signal was 0.25 MHz (the entire calibration procedure is described in the SpinAligner manual).

For a finer calibration of the carbon resonance frequency, ~ 22 mg of the pyruvate-radical concentrate (30 mM trityl-radical and 14 M 1-^13^C-PA) was filled in the sample cup and lowered to 10 mm above the bottom of the probe. After adjusting the LC circuit, DNP was commenced using continuous wave microwave irradiation ($p_{a}^{MW}$ = 16 mW and *ν*^**µW**^ = 187.135 GHz), and solid state ^13^C NMR signal was detected at *ν*^13C^ = 71.492 MHz (4 averages, the same RF parameters), resulting in a *B*_0_ = 6.6765 T and *ν*^e−^ = 187.17 GHz. ^13^C-FWHM was 0.16 MHz.

#### Calibration of sample position

**Figure 3:**
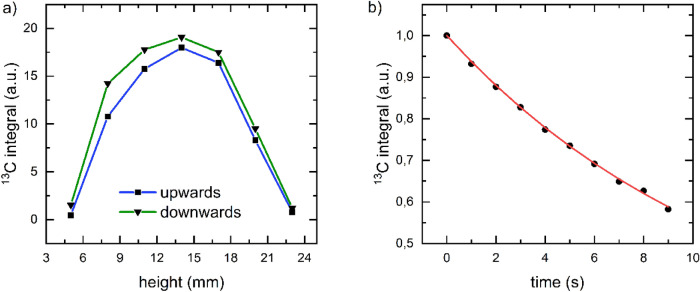
Calibration of the sample position and flip angle. (**a**) DNP-enhanced, solid-state ^13^C-NMR signal of pyruvate as a function of the position of the sample in probe. To ensure sufficient signal, the sample was first polarized for 40 min. Then the ^13^C signal was acquired at different positions x_s_ every minute using a low flip angle of 2°. Moving the sample first down, then up, a broad maximum around x_s_ = 14 mm was found. Straight lines were plotted to guide the eye. (**b**) DNP-enhanced ^13^C NMR signal acquired by a train of $p_{d}^{RF}$ = 2 us, $p_{a}^{RF}$ = 18 dB pulses with TX = 217 µs, TR = 1 s, NS = 1, NX = 49. By fitting a mono-exponential function to the data (red line, $\tau =$ 13.3 s, Eq. 1), the flip angle was determined to α = 3.2° (Eq. 2).

The sensitive area of the NMR coil was determined by acquiring the solid state, DNP-enhanced ^13^C-signal as function of the sample’s position (Fig. 3a). A broad maximum was found around x_s_ = 14 mm measured from the lowest position (bottom of probe). To assure a low sample temperature, we chose x_S_ = 10 mm for all following experiments (within 90% of the maximum signal).

#### Calibration of RF power

To calibrate the ^13^C flip angle, a standard sample was polarized by DNP and a train of low-angle free induction decays (FID) was acquired after turning off the microwaves ($p_{d}^{RF}$ = 2 us, $p_{a}^{RF}$ = 18 dB, TX = 217 µs, TR = 1 s, NS = 1, NX = 49 leading to N = 49 s^−1^ Fig. 3b). By fitting Eq. (1) to the signal, signal decay constant $\tau =$ 13.3 s and α = 3.2° was obtained using Eq. (2). For many of the following experiments, a flip angle of ~ 0.32° was used ($p_{d}^{RF}$ = 2 us, $p_{a}^{RF}$ = 38 dB).

#### Microwave calibration

**Figure 4:**
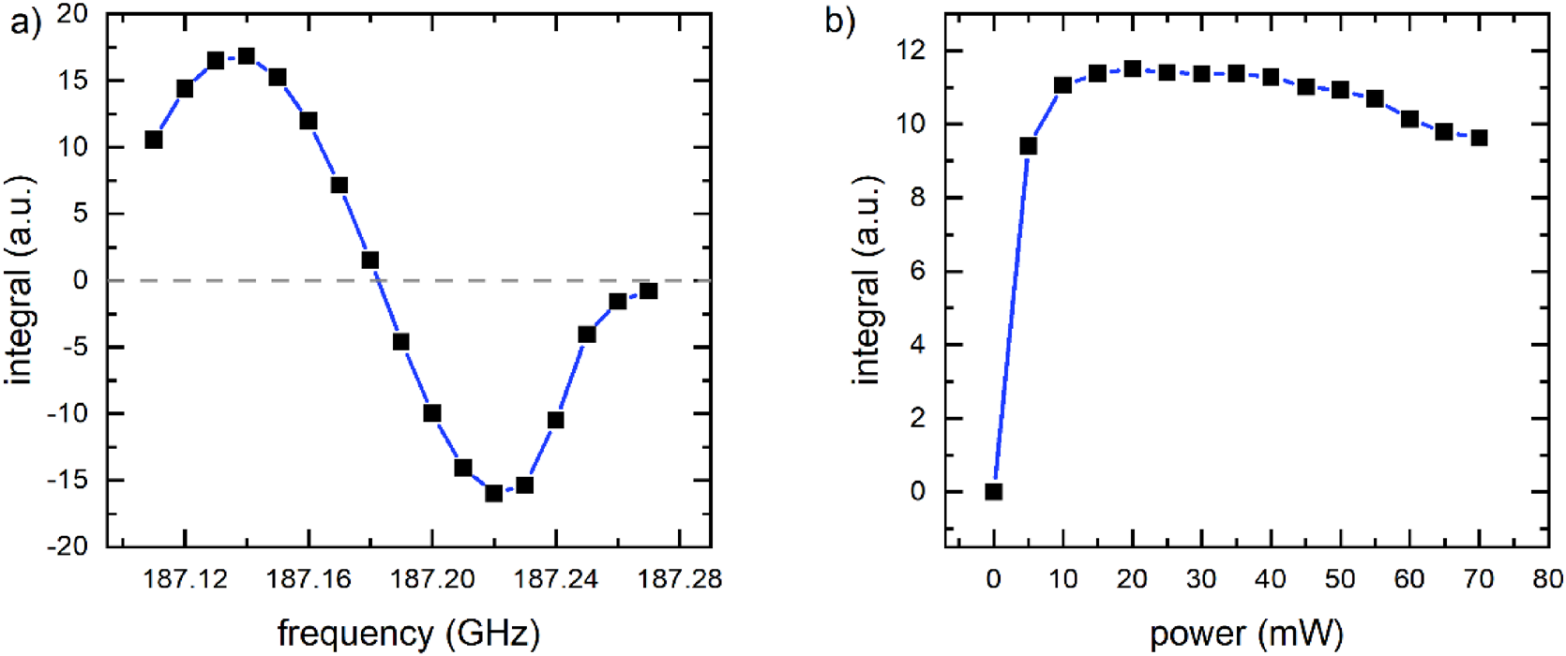
Optimization of ^13^C polarization transfer. DNP-enhanced, solid state ^13^C-NMR signal of pyruvate at ≈ 1.4 K as a function of microwave frequency (a, $p_{w}^{MW}$ = 30 mW) and microwave power (b, *ν*^MF^ ≈ 187.135 GHz). When the frequency was varied (a), two extrema were observed, and the first maximum at ≈ 187.135 GHz was chosen for later experiments. For the power sweep, the signal was found to increase up to 20 mW; 16 mW was used in the subsequent experiments. Straight lines were added to guide the eye. ^13^C polarization was destroyed after each signal acquisition. Each data point corresponds to the ^13^C-signals acquired after 2 min of DNP. NMR acquisition parameters were $p_{w}^{RF} =$ 2 µs, $p_{a}^{RF} =$ 3 dB, NS = 4, and α ≈ 18°.

To optimize DNP, the polarization transfer from electrons to nuclei, the DNP-enhanced, solid state, ^13^C-NMR signal of pyruvate-radical concentrate was acquired using TR = 2 min as a function of **µW** frequency (Fig. 4a, 10 MHz steps, $p_{a}^{RF} =$ 3 dB, NS = 4, with α = 18°) and fixed power $p_{w}^{{{\mathbf{\mu W}}}}$ = 30 mW. Two extrema were found. The maximum at 187.135 GHz was chosen for the following dDNP experiments. After each acquisition, the polarization was saturated with a train of 1000 pulses with 18° excitation angle.

**Figure 5:**
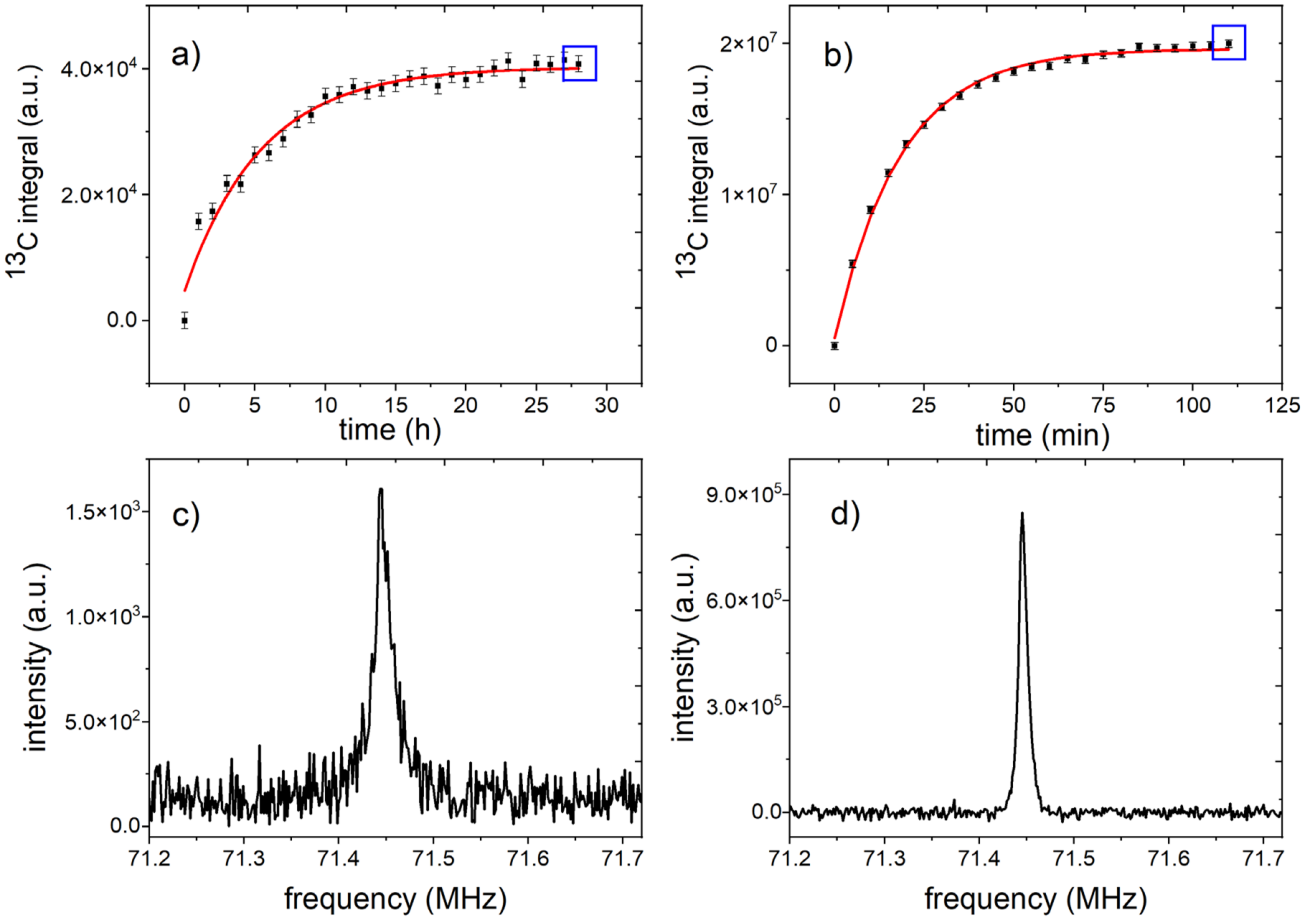
Quantification of solid-state polarization. Solid-state, ^13^C-NMR signals of 14 M 1-^13^C pyruvic acid mixed with 30 mM radical (112.3 mg total sample weight) monitored with low flip angle excitations ($\alpha$ ~ 0.32°, NS = NX = 256, TR = 1 h (a) or TR = 5 min (b)) while reaching thermal equilibrium at ≈ 1.4 K and 6.7 T (**a**) and during DNP (**b**). Blue squares indicate the spectra shown below (**c**, **d**). By fitting a mono-exponential recovery function (Eq. 5) to the polarization build up without **µW** (**a**) and with **µW** (**b**) and correcting for the RF excitations (Eq. 6), a solid-state relaxation $T_{1}^{ss} { } \approx { }\left( {5.45{ } \pm { }0.44} \right){\text{h }}$, signal at thermal equilibrium ${\text{signal }}S_{inf}^{ssTP } { } = { }\left( {4.07 \cdot 10^{4} \pm 0.1 \cdot 10^{4} } \right)$, DNP build-up time T^DNP^ = (18.396 ± 0.49) min and equilibrium signal $S_{inf}^{ssDNP}$ = ($2.0 \cdot 10^{7}$ ± $0.2 \cdot 10^{6}$) a.u. were obtained (note that this build up is faster than for the standard sample). The polarization of the spectrum acquired after 110 min DNP was quantified to $P^{obs,ssDNP}$ (110 min) = $S_{ }^{ssDNP}$(110 min) $P^{{{\text{TP}}}}$/$S_{inf}^{ssTP }$ ≈ 64%. Without RF excitations the expected steady state signal is estimated to be $P_{inf}^{ssDNP}$ = $S_{inf}^{ssDNP}$ $P^{{{\text{TP}}}}$/$S_{inf}^{ssTP }$ ≈ 61% The spectra (**c**) and (**d**) are the last measured thermal recovery and DNP spectra (marked on (**a**) and (**b**) with blue rectangles). The first six datapoints in (**a**) were neglected for the fit because of very low SNR.

To calibrate the **µW** power, we repeated the experiment, keeping frequency constant and varying the power (Figs. 4b, 5 mW increments). The ^13^C signal was found to increase steeply between ≈ 5 and 15 mW, forming a slowly declining plateau that decreased for powers larger than ≈ 40 mW. To avoid heating the sample by **µW** irradiation, while maintaining a high polarization, we chose a power of 16 mW for the following dDNP experiments.

### DNP performance

#### Solid-state polarization of 1-^13^C-pyruvate

The pyruvic acid-radical concentrate was filled into the cup and lowered to x_s_ = 10 mm above the bottom of the VTI at ≈ 1.4 K. While the sample was approaching the thermal equilibrium at *B*_0_ ≈ 6.7 T and T ≈ 1.4 K, thermal ^13^C signal was monitored for 24 h (Fig. 1). An asymptotic signal increase was observed, and a mono-exponential recovery function (Eq. 6) fitted to the data yielded an apparent solid-state relaxation time $T_{1}^{obs,ss}$ = (4.98 ± 0.40) h and apparent steady state thermal signal $S_{inf}^{obs,ssTP}$ = 3.9 × 10^4^ (R^2^ = 0.969). Using the NMR acquisition parameters ($\alpha$ ~ 0.32°, NS = NX = 256, and TR = 1 h) and Eqs. (3) and (6), the life-time corrected for the excitation was estimated to be $T_{1}^{ss}$ ≈ (5.45 ± 0.44) h (Eq. 6, N = 256/3600 s^−1^) and thermally polarized signal $S_{inf}^{ssTP }$ = 4.07 × 10^4^ (R^2^ = 0.97) (Eq. 7). At the end, the thermal polarization was saturated with a train of 1000 pulses with 5° flip angle.

Next, DNP was commenced by turning on the microwave source in continuous wave mode using the above described optimized settings. The build-up of the DNP enhanced ^13^C signal *S*^*DNP*^(t) was monitored by acquiring an NMR spectrum every minute (Fig. 1, the same parameters as before but with TR = 1 min). The last spectrum, acquired *t*^DNP^ = 110 min from the beginning of DNP, yielded a solid state polarization of P^obs,ssDNP^ = 64%. A mono-exponential recovery function was fitted to the data, yielding an apparent time constant of T^obs,DNP^ = (18.39 ± 0.50) min. The RF excitations almost did not affect the build-up of DNP signal: T^DNP^ = (18.396 ± 0.49) min, that corresponds to polarization of ≈ 61%.

#### Dissolution and quantification of liquid state ^13^C-polarization

A standard sample was polarized as described above with a 22 mg sample. Once the desired polarization was reached, the dissolution medium (specific to the tracer and sample size) was filled into a heating chamber (Fig. 1a-1). The solution was heated until a pressure of 11 bar was reached, corresponding to temperature T ≈ 115 ºC. Right before the medium was injected into the sample cup, the cup was lifted 8 cm to reduce the impact of the hot solution on the helium bath in the VTI. The injection of the dissolution medium into the sample cup was commenced via the polarizer’s software. Within ca. 2 s, the sample was dissolved and transferred into the receiver vessel through a double-walled tubing assembly (injection via inner tube, ejection via outer tube). As hot and pressurized liquids were involved, care was taken and safety measures applied.

**Figure 6:**
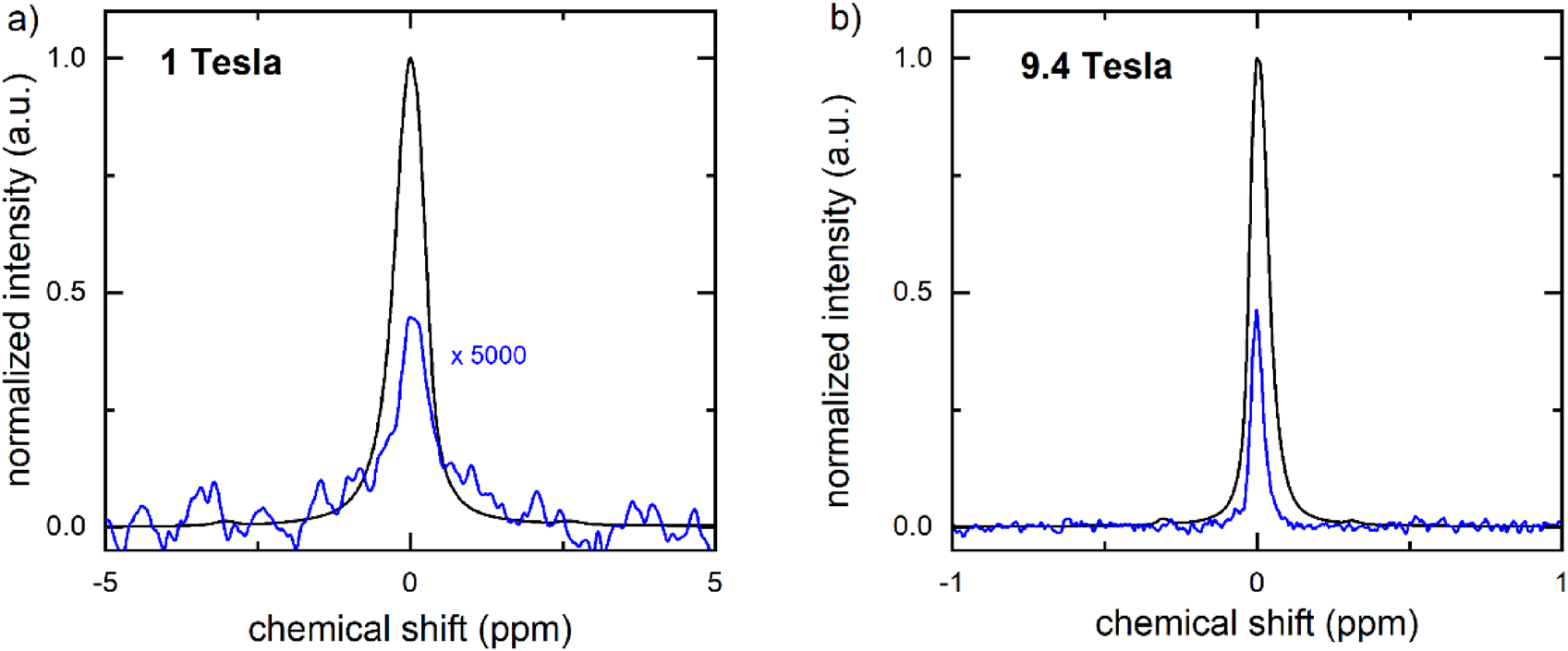
High resolution ^13^C-NMR spectra of hyperpolarized (black) thermally polarized (blue) 1-^13^C-PA measured at 1 T (**a**) and 9.4 T (**b**). (**a**) The hyperpolarized signal was measured in a single scan (NS^DNP^ = 1) after approx. 26 s after dissolution ($\alpha$ = 5°, p_w_ = 3.05 µs, p_a_ =  − 5.6 dB, S^DNP^ = 32.38 a.u.). The thermally polarized signal was acquired adding 4 vol% Gd contrast agent ($\alpha$ = 20°, p_w_ = 12.20 µs, p_a_ =  − 5.6 dB, S^DNP^ = 4.18 × 10^−4^ a.u.). The resulting signal enhancement to S^DNP^ was 1.09 × 10^9^ (Eq. 8), and the polarization = 26% (Eqs. 9, 10). (**b**) At 9.4 T, the signal was acquired ≈ 30 s after dissolution using a 5° pulse (S^DNP^ = 7.59 × 10^5^ a.u., p_w_ = 10 µs, p_a_ =  − 18.9 dB, RG = 0.25) and quantified with respect to the thermally polarized signal acquired with a single 90° pulse (S^TP^ = 1.41 × 10^5^ a.u., p_w_ = 0.55 µs, p_a_ = − 18.9 dB, RG = 101) to an enhancement of 2.5 × 10^4^ (Eq. 8) and polarization of 20% (Eqs. 9, 10). Note that due to the differences in the RG, both hyperpolarized spectra were normalized to 1 and the thermal spectrum measured at 1 T was multiplied by 5000 to fit in the scale.

To quantify the liquid state polarization, the sample was split between 5 mm NMR tubes and transferred manually to 1 T and 9.4 T NMR spectrometers, where the hyperpolarized and (later) thermally polarized signals were acquired (Fig. 6). In this example, the polarization was quantified to 26% at 1 T, 26 s after dissolution, and 20% at 9.4 T, 30 s after dissolution. The lifetimes of the polarization were measured to 67 s for 1 T and 48 s for 9.4 T. Using the longest T_1_ which also corresponds to the lower field, we estimated the polarization at the time of dissolution to be 38% for the sample measured at 1 T, and ≈ 31% for the 9.4 T sample. Note that the sample was exposed to different, varying and much lower magnetic fields during the transfer, so that using the high-field T_1_ to estimate the polarization only provides a very rough estimate.

#### Standard operational procedure, reproducibility and hyperpolarization yield

While performing more than 100 DNP experiments, the following procedure proved to be instrumental to obtain reproducible polarization for 1-^13^C-pyruvate. In addition to the initial calibrations during the setup (Table 2) we developed a more elaborate procedure which contains routine calibrations (Table 3), specific preparation of chemistry (Table 4), a 21-step polarization procedure (Table 5), and a weekly maintenance routine (Table 6). These procedures were developed for pyruvate polarization, but may serve as a starting point for other agents (e.g. below for ^15^N-urea), although some modifications will be necessary.

**Table 3:** Routine DNP procedure conducted before and during each DNP experiments.

Step	Parameter	Comments	Standard settings
1	Fluid path	Flush the sample path before inserting the sample	
2	^13^C transmitter frequency	Compare to calibrated value. Coincides with the center of the spectrum	± 10 kHz to calibrated value; adjust ^13^C and **µW** frequencies if deviation is larger
3	^13^C resonance frequency	Compare to transmitter frequency	
4	Resonance of the coil	Adjust variable capacitors if necessary	Reflection less than 10%
5	**µW** frequency, power and mode		Calculate **µW** maximum frequency from ^13^C resonance frequency. Constant **µW** mode
6	Buildup rate	Observe for abnormalities. Analysis mode should be set to integral	
7	Monitor SS signal	Check for good SNR

**Table 4:** Standard procedure to prepare pyruvate samples.

Step	Operation	Duration	Comments	Standard settings
1	Take one aliquot of around 1.5 mL of 1-^13^C-PA and one aliquot of around 50 mg of trityl radical out of the fridge			Before use store at − 20 °C
2	Wait until the pyruvic acid warms up and becomes liquid	20 min	Keep the vial in a warm place. Avoid direct sunlight	
3	Put 25.8 mg of trityl radical in a vial			e.g. Eppendorf tubes
4	Add 0.68 g of 1-^13^C-PA into the vial			
5	Mix it	10 min	Centrifuge the vial	
6	Divide into c.a. 250 µL aliquots			e.g. Eppendorf tubes
7	Put in the freezer			Store at − 20 °C

**Table 5:** Standard operational procedure for dDNP experiments.

No	Operation	Duration	Comments	Norm values
1	Check T of VTI (P1, P2)			T = 1.38–1.4 K P1 ≈ 1.3 mBar P2 ≈ 250–350 mBar
2	Warm up stock CA-radical concentrate until liquid	10 min	Protect from light	
3	Flush fluid path with air	10 min	Automatic	
4	Add CA-radical concentrate to cup	5 min	Use micropipet, add to the bottom, avoid smudging to walls	18–19 µL ≈ 22 mg (for ^13^C) And 50 uL ≈ 50 mg (for ^15^N)
5	Prepare liquid nitrogen (lN_2_)	2 min	Place non-magnetic dewar next to the magnet	< 500 mL of lN_2_
6	Connect sample cup to polarizer: - Connect empty cup - Flush with He - Disconnect cup, connect cup with a sample - Dip cup in lN_2_ - Pressurize fluid path	3 min	Mark vial to avoid damage to thread by over tightening Use cryogenic gloves Immerse slowly into lN_2_ Pressurize while in lN_2_	Immersion into lN_2_ for 30 s
		20 s		
7	Insert cup into VTI	5 min	Hurry but stay calm: open airlock, remove plug, insert sample, close airlock, start automatic insertion procedure Manually guide tube if needed. Use gloves	
8	Position cup in VTI/NMR coil		Mark lowest position on tube e.g. with tape	10 mm above lowest position
9	Check that the NMR coil is in resonance	2 min	Adjust tuning and matching capacitors	Tune within ± 20 kHz, match 1–2%
10	Wait for temperature T and pressure to stabilize (P1, P2)	5 min	Insertion causes temperature and pressure increase	Close to starting temperature, e.g. T = 1.38 – 1–4 K
11	Start DNP 11a: start monitoring 13C NMR (SpinIt) 11b: start DNP (SpinAligner)	60 min	Check that **µW** is on Check ^13^C NMR signal	
12	Prepare MRI/NMR		Adjust resonance of the probe, create a new protocol, shims of the system, create new project, free space to ease transport	
13	Inject dissolution medium in heater		Standard dissolution medium suitable for tracer	5 mL
14	Initiate heating of dissolution medium	5 min	Close the cover of polarizer	Automatic, ready when 11 bar reached
15	Prepare receiver vessel/syringe		Weigh and label NMR tubes and place receiver in the stray field	
16	Put on safety equipment: Goggles, gloves		Caution! Hot liquids, high pressures!	
17	Raise cup	30 s	Raise cup above lHe level to reduce lHe boil off	Rise by 8 cm
18	Execute dissolution	< 30 s	Hot liquid at high pressure and speed will be ejected	Automatic
19	Collect hyperpolarized medium e.g. to syringe and apply	5 s	Draw in syringe for manual injection, transfer via tubes to detection site	
20	Transfer NMR samples or syringe to NMR/MRI and execute pulse sequences when sample is at the detection position	30 s	A fast transfer is necessary	
21	Take out the sample cup, conserve the airlock, perform the cleaning	15 min	Wash the heater 3 times with deionized water, then dry at least 10 min	10 mL of water to clean the system each time

**Table 6:** dDNP maintenance routine, conducted once a week (automatically and scheduled).

No	Operation	Comments	Norm values
	Stop the dry pump		0 Hz
	Reduce the He flow		
	The VTI T increases automatically	It takes at least 6 h	150 K (at least)
	The system stays warm for at least 38 h	It is possible to use the entire weekend	
	Automatic cleaning of the VTI by using the vacuum pump when the T is higher than 150 K		
	Decrease the T again	~ 3 h	

The total duration is 48 h from the start of the routine.

Using these procedures, we evaluated the reproducibility and yield for obtaining ≈ 4 mL solution with 60 mM hyperpolarized pyruvic acid, a composition suitable for animal experiments^30–32^. The dDNP process was repeated five times on different days using standard samples of (21.64 ± 0.15) mg and 3.9 mL dissolution medium and detection at 1 T (Table 7). On average, we obtained a build-up constant **T**^**DNP**^ = (1032 ± 21.7) s and solid state polarization of (42.1 ± 3.7) a.u.. Depending on the transfer time t^trans^ (17–20 s), the liquid state polarization was quantified with respect to the thermally polarized sample to *P* ≈ 33%–46%, with a mean of (38 ± 5.7) %. To estimate the polarization right after dissolution, we used the T_1_ of the samples and obtained an average polarization of (47.2 ± 7.8) %^33^. The pH value of the sample inside the NMR tube was measured to be 8.51 ± 0.02.

**Table 7:** Reproducibility of hyperpolarized ^13^C pyruvate in aqueous solution as contrast agent for MRI.

No	c(^13^C) (mM)	T^DNP^ (s)	t^trans^ (s)	$S^{lsHP}$(a.u)	$S^{lsTP}$/10^6^ (a.u)	ε	P(t^trans^) (%)	P(0) (%)	T_1_ (s)	pH
1	61.19	1000	19	46.2	450	400,206	34	42.3	88	8.40
2	59.51	1047	19	37.2	380	385,863	33	39.6	101	8.32
3	58.39	1018	20	41.1	380	421,095	36	44.8	89	8.90
4	59.23	1048	20	46.5	330	547,196	46	57.5	94	8.77
5	56.14	1045	17	39.5	310	502,997	43	51.9	87	8.65
**M**	**58.89**	**1032**	**19**	**42.1**	**370**	**451,471**	**38**	**47.2**	**92**	**8.60**
***c***_**v**_	**3.1%**	**2.1%**	**6.4%**	**8.8%**	**13.1%**	**13.9%**	**15.0%**	**15.6%**	**6.3%**	**2.8%**

Mean and coefficient of variance (*c*_v_) for concentration of 1-^13^C-PA after the dissolution, fitted time constant of the hyperpolarization build-up (T^DNP^), transfer time ${t}^{trans}$ to 1 T NMR, liquid-state NMR signal of hyperpolarized sample *S*^*lsHP*^ normalized to the largest signal (sample 4), liquid-state NMR signal in thermal equilibrium (${S}^{lsTP}$) normalized to the largest signal (sample 1), polarization at the time of measurement **P(t**^**trans**^**)**, estimated polarization directly after the dissolution **P(0)** and lifetime (T_1_) of hyperpolarization at 1 T (the enhancement was calculated using flip angle α = 5º, RG = 31, ns = 1 for hyperpolarized sample, and α = 20º, with a RG = 31, and ns = 3600 for the thermally polarized sample). Significant values are in [bold].

#### dDNP duty cycle

In addition to reproducibility experiments, to evaluate how many samples can be polarized in a given time, we performed seven more 1-^13^C-PA DNP experiments every ≈ 90 min using the procedure described above: roughly 30 min were needed to prepare and clean the system, and ca. 60 min for dDNP. On average, measured liquid state polarization *P*(**t**^**trans**^ ≈26 s) = (33 ± 3.3) % was achieved that corresponds to the estimated value right after dissolution *P*(**t**^**trans**^ = 0) = (53.9 ± 12.4) %.

#### In vivo ^13^C MRI

To demonstrate the feasibility of metabolic imaging in vivo, we polarized 1-^13^C-PA with the procedure described above to *P*(**t**^**trans**^ = 0) ≈ 50%.

**Figure 7:**
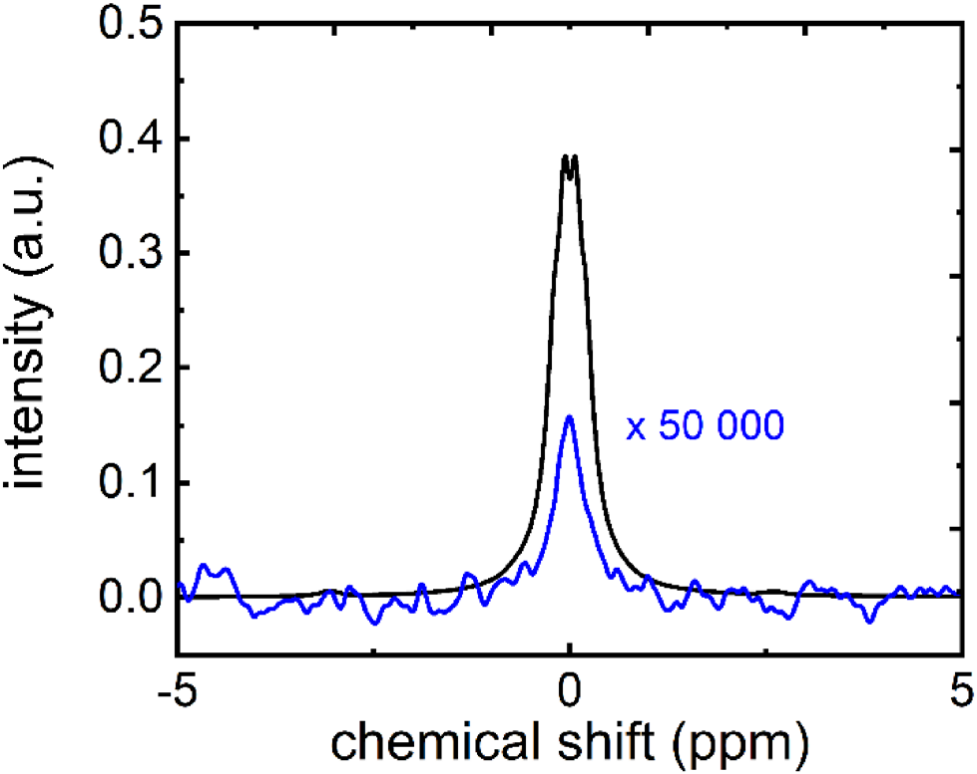
High resolution ^13^C-NMR spectra of hyperpolarized (black) thermally polarized (blue) 1-^13^C-PA (sample 5 in the table) measured at 1 T. The hyperpolarized signal was measured in a single scan (NS^DNP^ = 1) after approx. 17 s after dissolution ($\alpha$ = 5°, p_w_ = 3.05 µs, p_a_ = − 5.6 dB). The thermally polarized signal was acquired adding 4 vol% Gd contrast agent ($\alpha$ = 20°, p_w_ = 12.20 µs, p_a_ = − 5.6 dB, S^DNP^ = 39.5 a.u.). The resulting signal enhancement to S^DNP^ was 310 × 10^6^ (Eq. 8), and the polarization = 43% (Eqs. 9, 10). The thermal spectrum measured was multiplied by 50,000 to fit in the scale.

During the buildup of the polarization, a CEBAC-mouse was anesthetized, outfitted with a tail-vein catheter, and placed on the heated animal bed of the 7 T MRI system. The MRI coil was adjusted and anatomical images were acquired before dissolution (Fig. 7). No tumor was apparent on the conventional MRI.

**Figure 8:**
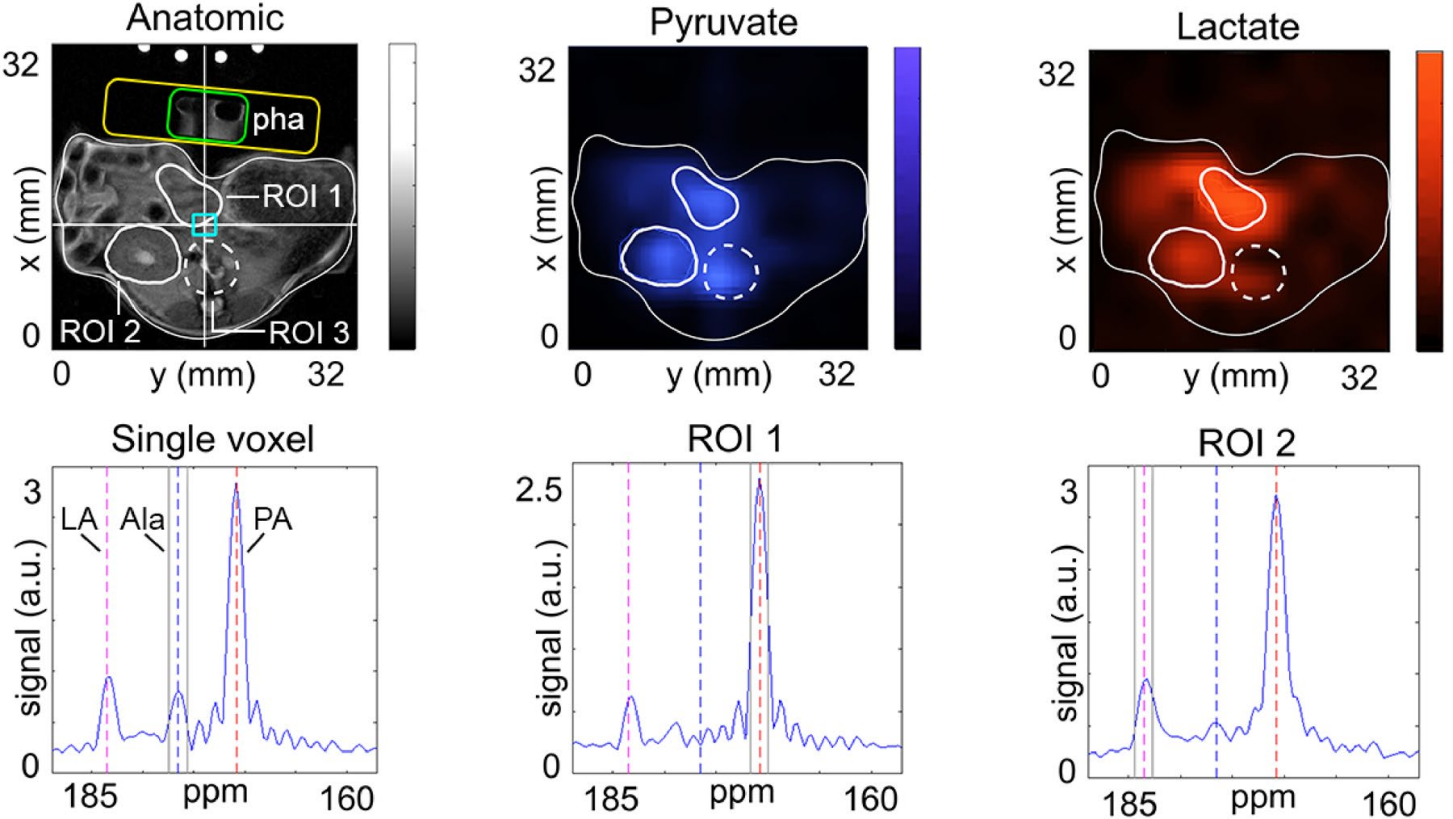
In vivo T1w ^1^H-MRI, maps of hyperpolarized 1-^13^C-PA and 1-^13^C-lactate (LA) and selected ^13^C spectra of a pancreatic tumor rat model acquired at 7 T. After the injection of 100 µl hyperpolarized contrast agent containing ~ 46 mM 1-^13^C-PA, eight CSI datasets were acquired. Prominent signal of lactate was found in the regions of kidney (ROI1), liver (ROI2), *aorta* and inferior *vena cava* branch (ROI3). Strong SNR was observed in the selected voxel (blue rectangle), exhibiting resonances of lactate, pyruvate and alanine (Ala) with a line width of ca. 84 Hz. The receive-only loop-coil was positioned ventral (yellow rectangle) and a small container filled with water was placed in the middle of the coil (green rectangle, phantom—Pha). Note that the tubes supplying warm water to the animal bed appeared on the top because of aliasing. The sum of eight consequent acquisitions is shown.

After the dissolution with 5 mL medium, the contrast agent containing approx. 46 mM 1-^13^C-PA was rapidly transferred to the MRI and ≈ 100 uL were injected into the tail vein catheter of the mouse within 40 s after dissolution. About 10 s after the end of the injection, ^13^C-CSI was performed across an abdominal axial slice. Signals of pyruvate, lactate and alanine were observed, and maps of each metabolite were prepared (Fig. 8). Using the same geometry ^1^H T1w MRI was measured. We observed strong lactate and pyruvate signals in the liver and kidney. High SNR was observed in individual voxels as well in larger ROIs.

#### dDNP of other nuclei: demonstration with ^15^N

Exchanging the LC box allowed us to set the resonance frequency of the NMR coil in the polarizer to ≈ 28.83 MHz, the resonance frequency of ^15^N at 6.7 T. This allowed us to calibrate the ^15^N flip angle, **µW** frequency and power using a 200 mg sample of 4.16 mM ^13^C,^15^N_2_-urea and 35.7 mM radical (α = 3.5° at *p*_w_ = 3 us, *p*_a_ = 10 dB, ν(**µW**) = 187.18 GHz, p(**µW**) = 35 mW). DNP-enhanced solid state ^15^N-NMR signal was readily observed, and a build-up constant T^DNP^ = (2140 ± 391) s was obtained for ^13^C,^15^N_2_-urea and (3358 ± 425) s for ^15^N_2_ urea.

Dissolution was performed using 5 mL of deionized H_2_O and 0.27 mM EDTA, leading to a nominal 45.4 mM urea concentration after dissolution. After transfer to the 9.4 T NMR spectrometer, sixty 5° ^15^N-spectra were acquired (TR = 3 s), and used to calculate T_1_ and quantify the polarization. The lifetime of the hyperpolarized sample was determined to T_1_(^13^C-^15^N urea) = (26.3 ± 1.0) s and T_1_(^15^N-urea) = (26.1 ± 0.7) s.

For ^13^C-^15^N_2_ urea, a ^15^N polarization of (4.5 ± 0.7) % was observed *t*^trans^ = (30 ± 1) s after dissolution (n = 4) and for ^15^N_2_-urea, the polarization was determined to (5.6 ± 0.8) % after (30 ± 3) s (n = 4), both with respect to the ^15^N-signal of each sample in thermal equilibrium (TR = 17 s, 128 averages, RG 101, α = 90°). The initial ^15^N-polarization at the time of dissolution (t^trans^ = 0 s) was estimated to P(^15^N) (t = 0 s) = (14.7 ± 1.7)% for ^13^C-^15^N_2_ urea and P(^15^N) (t = 0 s) = (18.4 ± 1.1)% for ^15^N_2_ urea^34^.

## Discussion

In this paper, we describe our initial experience, operational routines and performance of a cryogen-free dDNP polarizer operated at 6.7 T.

### Installation requirements

The polarizer requires ≈ 2–3 m^2^ footprint, standard single-phase electric power, compressed air and helium. The magnet is not actively shielded so safety has to be considered and some distance from other devices must be maintained. The helium compressor requires 3-phase power and cooling water;—prerequisites that are likely met by many NMR or MRI facilities. The noise of the helium pump at the polarizer and the cryo-expander may be an inconvenience for those working nearby for an extended time.

### Safety

The main hazards of the setup include hot and cold pressurized fluids: water at 115 °C and 11 bar, liquid nitrogen, max. 2 bar helium and compressed air, acids and bases, magnetic fields (6.7 T) and electricity (230 V). In addition, the pressurized helium lines, standard gas bottles (e.g. 200 bar) as well as any chemical hazards have to be considered. The dissolution (heating, pressurization and extraction) takes place in a well-controlled manner behind closed, transparent doors, so that splash and splinter protection is provided (although no such event occurred). After the dissolution, the medium has sufficiently cooled to ≈ 37 °C, if necessary a jar of ice can be placed beneath the receiver vial. Cold temperatures are present while freezing the sample in liquid N_2_, and precautions include using appropriate safety gear (gloves, googles).

As the magnet contains no liquid cryogens, the safety precautions can be adjusted accordingly. A quench pipe is not needed, as the amount of gaseous helium used to cool the VTI is only 50 standard liters although there is more helium in the closed-cycle He-cryostat.

### Handling and operation

The system provides full access to more than 4 sensor readouts, all of which are continuously stored for later retrieval. We found this to be an excellent and essential feature, allowing precise documentation and reconstruction of the experimental conditions. The software interface (LabView) provides control over many essential DNP parameters, and new features may be added by the user or manufacturer. It should be noted, however, that the temperature of the sample itself cannot be directly measured, and is estimated by measuring the temperature of the VTI.

Most parts are easily accessible for repairs or modifications, e.g. to adjust the amount of hyperpolarized substance, or to supply filtered air to the enclosure. As most valves are operated by compressed air, no heating and melting of the valves was observed.

Switching between different nuclei by tuning or exchanging the LC circuity was very convenient for monitoring the hyperpolarization of different nuclei.

### Reproducibility

Using the polarization routines described above, robust and reliable liquid state hyperpolarization was achieved: P(^13^C ) with (*t*^trans^ = 19 s ± 1 s) = (38 ± 6)%, and an estimated polarization at dissolution of P(^13^C ) at t_0_ ≈ (47 ± 7)% (Table 7). Since, the actual decay of the sample during the transfer across strongly varying magnetic fields is not precisely known, the polarization at the time of dissolution is an estimate. The build-up of the solid-state polarization yielded a time constant of ~ 17 min, allowing for repetitive dissolutions with a duty cycle below two hours, which may be accelerated further if the need arises. The reported data were acquired after the level of the liquid helium in the VTI was raised by submerging a disk into the liquid. This modification resulted in less variable build-up times: *c*_v_(T^DNP^) = 2.1% and 22.8% with and without washer, respectively. It is relevant to point out that the temperature inside the VTI is not necessary the temperature of the sample inside the vial.

Interestingly, the ^13^C-NMR signal of pyruvate in the dissolved sample after hyperpolarization showed a relatively large variation, *c*_v_(S^lsTH^) = 13.1%. This result may indicate a varying pyruvate concentration, hinting at an inhomogeneous dissolution.

There is little data published with respect to reproducibility in the literature, but the absolute yield is comparable to what was reported before (1-^13^C-PA with trityl radical, i.e. 36–64%^35^). Of course, a faster and more reproducible sample transfer will improve the significance of these numbers; a dedicated delivery system will be presented elsewhere.

### Polarization of other nuclei

Tuning the NMR coil of the DNP to other nuclei was easy as the LC circuit was placed in a shielded box outside the VTI. For resonance frequencies close to ^13^C (like ^129^Xe), it was sufficient to adjust the variable capacitors. For other nuclei, a different LC circuit was used. We demonstrated this by using ^15^N urea, whose gyromagnetic ratio is about 1/10 of that of ^1^H and 40% of ^13^C, and obtained a polarization of 5.6% at the spectrometer.

### Pitfalls

During the course of this study, less than 5% of all experiments failed. Most of these were during the dissolution and before the cleaning procedures were in place. Among the issued encountered were:

#### Sample insertion failed

As the tubes for the dissolution have a smooth surface to assure a tight seal, the lowering mechanism was found to slip sometimes. This issue was easily addressed by gently assisting the insertion manually. A more serious problem occurred after three weeks of continuous using, when we experienced problems lowering the sample into the magnet. We assume that this was caused by ice starting to form inside the tube used to insert the sample, possibly caused by small leaks or the frequent insertion/ejection of the sample cup. The issue was resolved by warming up the VTI, so we implemented a weekly cleaning procedure (warming up the VTI to c.a. 180 K within ≈ 10 h and gas evacuation (1 h) by vacuum pump, followed by cooling to ≈ 1.4 K in ≈ 5 h). This procedure can be conducted automatically e.g. over the weekend.

#### Low solid-state polarization

To obtain consistent solid-state polarization, it turned out to be essential to freeze the sample in the cup (in liquid nitrogen) before inserting it in the magnet. This way, splashing of the sample during the pressurization was avoided, so that the sample stayed at the bottom of the cup. Furthermore, a mismatch of the (ESR and NMR) transmitter frequencies and the Larmor frequencies (caused by drifting of the magnetic field) may cause loss of polarization, so regular adjustments are needed. Additionally, it is relevant to underline the importance of keeping the sample vial in liquid He during the build-up, to assure the transfer of the highest electron polarization. Due to the relationship between hyperpolarization and T, any temperature higher than 3 K implies considerable losses in polarization.

#### Failed dissolution

To avoid any faulty dissolution, it was essential to dry the dissolution module two times by flushing all the tubes with compressed air for 7–10 min before starting the polarization procedure by inserting the sample. As the tubes and connectors for dissolution were exposed to highly varying temperatures and pressures, regular maintenance is required. In more than 100 dissolutions, we observed one material failure of the dissolution path so far (broken tube, unclear reason).

#### Varying liquid state polarization

Obviously, varying times and magnetic fields during transfer will cause varying polarizations. We ameliorated this issue by filling the NMR tubes on top of the magnet, where the field is ~ 10 mT, and by using a 12 mT resistive magnet to transfer the sample to the 9.4 T NMR. Using low-field NMR spectrometers to detect the enhanced ^13^C signal is advantageous as they can be placed close to the DNP system. However, quantification of the polarization is more difficult as the sensitivity is limited, requiring use of gadolinium relaxation agent and massive averaging to acquire sufficient SNR of the thermal ^13^C (no signal was observed for ^15^N).

## Conclusion

In this paper, we share our experience, results and tips for operating the SpinAlinger polarizer of more than 100 experiments over the course of one year.

Overall, the polarizer turned out to be reliable, compact, easy in use and providing high polarization. Other advantages include the absence of liquid cryogens, a short duty cycle and an open, modular design that e.g. allows monitoring of solid-state signals of different nuclei. We estimated the liquid state polarization of 1-^13^C-PA after dissolution to (38 ± 5.7) % and ^15^N_2_-urea to (5.6 ± 0.8) %—well sufficient for metabolic imaging as was successfully demonstrated here.

## Supplementary Information

Supplementary Information 1.

## Data Availability

The datasets generated and/or analyzed during the current study are available in the Zenodo repository, at the following https://doi.org/10.5281/zenodo.5957503.
