# Supplementary material for: Performance and reproducibility of 13C and 15N hyperpolarization using a cryogen-free DNP polarizer
Source: Sci Rep. 2022 Jul 8;12:11694. doi: 10.1038/s41598-022-15380-7 (PMC9270333; doi:10.1038/s41598-022-15380-7)
Supplement: Supplementary file 1 — Supplementary Information 1. [file 41598_2022_15380_MOESM1_ESM.docx]

Supporting materials: “Performance and reproducibility of ^13^C and ^15^N hyperpolarization using a cryogen-free DNP polarizer”

Arianna Ferrari,^a^ Josh Peters,^a^ Mariia Anikeeva,^a^ Andrey Pravdivtsev,^a^ Frowin Ellermann,^a^ Kolja Them,^a^ Olga Will,^a^ Eva Peschke,^a^ Hikari A. I. Yoshihara,^b^ Olav Jansen,^c^ Jan-Bernd Hövener ^a^

^a^ Section Biomedical Imaging, MOIN CC, Department of Radiology and Neuroradiology, University Medical Center Schleswig-Holstein, Kiel University, Germany

^b^ Institute of Physics, EPFL (Swiss Federal Institute of Technology), Lausanne, Switzerland

^c^ Department of Radiology and Neuroradiology, University Medical Center Schleswig-Holstein, Kiel University, Germany

ORCID Numbers:

Arianna Ferrari: 0000-0003-3593-9993

Josh Peters: 0000-0003-1019-4067

Mariia Anikeeva: 0000-0002-2443-9910

Andrey Pravdivtsev: 0000-0002-8763-617X

Frowin Ellermann: 0000-0001-6446-6641

Kolja Them: 0000-0002-5512-0910

Hikari Yoshihara: 0000-0002-3274-7147

Olav Jansen: 0000-0002-7330-1942

Jan-Bernd Hövener: 0000-0001-7255-7252

**Correspondence:**

Arianna Ferrari, M. Sc., PhD student

[arianna.ferrari@rad.uni-kiel.de](mailto:arianna.ferrari@rad.uni-kiel.de)

Jan-Bernd Hövener, Dr., Prof.

[jan.hoevener@rad.uni-kiel.de](mailto:jan.hoevener@rad.uni-kiel.de)

Contents

1. Gyromagnetic ratios 3
2. Theory 4

Magnetization relaxation, decay and build-up 4

1. Methods 7

NMR flip angle calibration equation 7

Liquid-state polarization decay 7

Solid state build-up evaluation 8

1. Results: Calibration of RF frequency and RF power 8
2. Gyromagnetic ratios

- Free electron, g-factor is 2.0023193, then its gyromagnetic ratio is

γ^free e^ = 176085.9644 x 10^6^ rad/(s·T), and γ^free e^ /2π = 28024.95164 MHz/T.

- The g-tensor for the OX063 trityl (AH111501 is the methylated OX063) was determined by Lumata et al^1^ to be axially symmetric with g_ꓕ_ = 2.00319(3) and g_ǁ_ = 2.00258 and hence g_iso_= (2*g_ꓕ_+ g_ǁ_)/3= 2.0029887, then its isotropic gyromagnetic ratio is γ^OX063^ = 176144.8288 x 10^6^ rad/(s·T), and γ^OX063^ /2π = 28034.3202 MHz/T.

The gyromagnetic ratio for some relevant nuclei are:

- γ^1H^ = 267.522208 x 10^6^ rad/(s·T), and γ^1H^ /2π = 42.577 MHz/T.
- γ^129Xe^ = -74.5210 x 10^6^ rad/(s·T), and γ^129Xe^ = 11.860 MHz/T.
- γ^63Cu^ = 71.11791 x 10^6^ rad/(s·T), and γ^63Cu^ /2π = 11.319 MHz/T.
- γ^13C^ = 67.28286 x 10^6^ rad/(s·T), and γ^13C^ /2π = 10.708 MHz/T.
- γ^15N^ = -27.126189 x 10^6^ rad/(s·T), and γ^15N^ /2π = 4.317 MHz/T.

1. Theory

**Magnetization relaxation, decay and build-up**

Bloch equation gives a good phenomenological description of relaxation of longitudinal magnetization Mz.

$$\frac{dM_{Z}}{dt}=\frac{M_{0}-M_{Z}}{T_{1}}=R_{1}\left( M_{0}-M_{Z} \right)$$

The general solution is

$$M_{Z}\left( t \right)=\left[ M_{Z}\left( t=0 \right)-M_{0} \right]e^{-R_{1}t}+M_{0}$$

With $M_{0}$ thermal magnetization, $R_{1}=\frac{1}{T_{1}}$ longitudinal relaxation rate.

Using chemical analogy, it can be written as two elementary reactions:

$$M_{Z}\overset{R_{1}}{\to}$$

$$M_{0}\overset{R_{1}}{\to}M_{Z}$$

We often used small flipping angle to observe signal decay or build-up. This can be modeled by additional “elementary reaction” step with $M_{Z}$decay with rate:

$$M_{Z}\overset{R_{\alpha}}{\to}$$

Then the corresponding joint differential equation is

$$\frac{dM_{Z}}{dt}=R_{1}M_{0}-\left( R_{1}+R_{\alpha} \right)M_{Z}$$

The general solution is

$M_{Z}\left( t \right)=\left[ M_{Z}\left( t=0 \right)-\frac{R_{1}}{R_{1}+R_{\alpha}}M_{0} \right]e^{-\left( R_{\alpha}+R_{1} \right)t}+\frac{R_{1}}{R_{1}+R_{\alpha}}M_{0}$ Eq. (1)

If $R_{\alpha}=0$ then it is identical to the general solution of unmodified Bloch equation.

If $R_{\alpha}\gg R_{1}$, then $M_{Z}\left( t \right)=M_{Z}\left( t=0 \right)e^{-\left( R_{\alpha}+R_{1} \right)t}$.

**Hyperpolarization decay:** $M_{0}=0$, $M_{Z}\left( t \right)=M_{Z}\left( t=0 \right)e^{-\left( R_{\alpha}+R_{1} \right)t}=Const\cdot e^{-R_{1}^{obs}t}$

Hence $R_{1}=R_{1}^{obs}-R_{\alpha}$.

**Build-up:** $M_{Z}\left( t=0 \right)=0$, $M_{Z}\left( t \right)=\frac{R_{1}}{R_{1}+R_{\alpha}}M_{0}\left( 1-e^{-\left( R_{\alpha}+R_{1} \right)t} \right)=Const\cdot\left( 1-e^{-R_{1}^{obs}t} \right)$

Again $R_{1}=R_{1}^{obs}-R_{\alpha}$.

Now we need only to see that is $R_{\alpha}$. Imaging that you apply equidistant RF pulses with angle α and N number of scans per second. Each pulse reduces the magnetization by $\cos\left( \alpha\right)$. Then in one second the reduction of magnetization is $\cos^{N} \left( \alpha\right)$, hence $e^{-R_{\alpha}\cdot1s}=\cos^{N} \left( \alpha\right)$ or $R_{\alpha}=-N\ln\left( \cos\left( \alpha\right) \right)$.

Therefore, the equation that we were looking for is

$R_{1}=R_{1}^{obs}+N\ln\left( \cos\left( \alpha\right) \right)$ Eq. (2).

Or in terms of T1:

$$T_{1}=\left( \frac{1}{T_{1}^{obs}}+N\ln\left( \cos\left( \alpha\right) \right) \right)^{-1}$$

When RF-pulses are equidistant with the interval TR, then N=1/TR and

$$T_{1}=\left( \frac{1}{T_{1}^{obs}}+\frac{\ln\left( \cos\left( \alpha\right) \right)}{TR} \right)^{-1}$$

**Taylor expansion of** $\boldsymbol{R}_{\boldsymbol{\alpha}}=-\boldsymbol{N}\ln\left( \cos\left( \boldsymbol{\alpha} \right) \right)$**.**

Usually, the angle $\alpha\sim5^{o}\sim0.087\ll1$. It means that one can use Taylor series:

$$\cos\left( x \right)\cong1-\frac{x^{2}}{2}+\frac{x^{4}}{4!}$$

$$\ln\left( 1+x \right)\cong x-\frac{x^{2}}{2}$$

And hence

$\ln\left( \cos\left( x \right) \right)\cong\ln\left( 1-\frac{x^{2}}{2}+\frac{x^{4}}{4!} \right)\cong\left( -\frac{x^{2}}{2}+\frac{x^{4}}{4!} \right)-\left( -\frac{x^{2}}{2}+\frac{x^{4}}{4!} \right)^{2}\cong-\frac{x^{2}}{2}+\frac{x^{4}}{4!}-\frac{x^{4}}{4}\cong-\frac{x^{2}}{2}-\frac{5x^{4}}{24}\cong-\frac{x^{2}}{2}$.

Hence for the small flipping angle excitations applied N times per second the effective signal decay rate is

$$R_{\alpha}=-N\ln\left( \cos\left( \alpha\right) \right)\cong N\frac{\alpha^{2}}{2}$$

For $\alpha=5^{o}$ and N~260 s^-1^, $R_{\alpha}\cong1$ s^-1^.

1. Methods

**NMR flip angle calibration equation**

Hyperpolarized signal decay induced by RF-pulses was fitted with a monoexponential decay function

$S^{obs}(t)=S_{0}\cdot e^{-t/\tau}$

After each excitation with angle $\alpha$ the signal decay by $cos(\alpha)$. After n pulses the initial signal $S_{0}$ is decayed by $\cos^{n} (\alpha)$. At each time point the signal then is given by

$S\left( t \right)=S_{0}ꞏ\cos^{\mathrm{tN}} \left( \alpha\right)$,

Where again N is the average number of excitations per second. Here the relaxation is completely neglected. Hence the angle can be estimated as

$\cos^{N} \left( \alpha\right)=e^{-1/\tau}$ or

$\alpha=arccos\left( e^{-\frac{1}{\tau N}} \right)$.

Note that it is not necessary to measure the spectrum after each excitation, however necessary to measure signal decay long enough for the most precise evaluation of decay constant $\tau$. Experimentally we measured often only each 10^th^ spectrum.

Note also that the same equation is immediately obtained also from Eq. (2).

Liquid-state polarization decay

Without signal excitation at the constant conditions the signal is expected to decay monoexponentially

$$S\left( t \right)=S_{0}\cdot e^{-\frac{t}{{T_{1}}^{HP}}}$$

From initial $S_{0}$ value to zero (thermal polarization is much smaller than $S_{0}$ and neglected).

However, because we use small flipping angle $\alpha_{HP}$ to track the slow signal decay, the observed decay constant is different

$$S^{obs}\left( t \right)=S_{0}\cdot e^{-\frac{t}{T_{1}^{obs}}}$$

The signal decays during interval TR=1/N by $e^{-\frac{TR}{T_{1}^{HP}}}$ and then by excitation by $\cos\left( \alpha_{HP} \right).$ It means that at each time point the signal is given by

$S^{obs}\left( t \right)=S_{0}\cdot e^{-\frac{t}{T_{1}^{obs}}}=S_{0}ꞏ\left[ e^{-\frac{TR}{T_{1}^{HP}}}\cos\left( \alpha_{HP} \right) \right]^{\frac{t}{TR}}$

And hence

$T_{1}^{HP}=\left( \frac{1}{T_{1}^{obs}}+\frac{\ln\left( \cos\left( \alpha_{HP} \right) \right)}{TR} \right)^{-1}$

This is equivalent to general solution given by Eq. (2).

**Solid state build-up evaluation**

When the signal recoveries from zero to thermal equilibrium it can be described by monoexponential recovery function

$S\left( t \right)=S_{inf}\left( 1-e^{-\frac{t}{T_{1}}} \right)$

Here $S_{inf}$ is the value at $t\to+\infty$. However, the function is different from the observations when series of small flipping angles are used to trace the signal recovery

$S^{obs}\left( t \right)=S_{inf}^{obs}\left( 1-e^{-\frac{t}{T_{1}^{obs}}} \right)$

To access $T_{1}$ value and $S_{inf}$ we suggest using Eq. (1)-(2). Then $T_{1}$ and $S_{inf}$ can be evaluated from $T_{1}^{obs}$ and $S_{inf}^{\alpha}$ as

$T_{1}=T_{1}^{obs}\left( 1+NT_{1}^{obs}\ln\left( \cos\left( \alpha\right) \right) \right)^{-1}\cong T_{1}^{obs}\left( 1-\frac{NT_{1}^{obs}\alpha^{2}}{2} \right)^{-1}>T_{1}^{obs}$ and

$S_{inf}=\left[ 1-NT_{1}\ln\left( \cos\left( \alpha\right) \right) \right]S_{inf}^{obs}\cong\left[ 1+\frac{NT_{1}\alpha^{2}}{2} \right]S_{inf}^{obs}>S_{inf}^{obs}$

When thermal equilibrium is discussed $S_{inf}\sim M_{0}$ – equilibrium magnetization value. We used these equation and method to describe the build-up of DNP and thermal polarization in the solid-state (SS).

1. Results: Calibration of RF frequency and RF power

We calibrated 13C flipping angle using $p_{a}^{RF}$ = 30 dB, $p_{d}^{RF}$ = 2 µs and get average flipping angle of 3.19^o^. For $p_{a}^{RF}$ = 10 dB and the same $p_{d}^{RF}$ = 2 µs the flipping angle would be approx. 32^o^. This was done and measured before the introduction of the additional RF power attenuator which reduces the power close to 4 times or by 12 dB. It means that with the current settings 32^o^ angle would corresponds $p_{a}^{RF}$ = -2 dB and the same $p_{d}^{RF}$ = 2 µs which is reported in the manuscript.

Also, we often use 0.31^o^ pulse which corresponds to $p_{d}^{RF}$ = 2 us, $p_{a}^{RF}$ = 50 dB with the old settings or $p_{d}^{RF}$ = 2 us, $p_{a}^{RF}$ = 38 dB with the attenuator.

We also used $p_{d}^{RF}$ = 2 us, $p_{a}^{RF}$ = 15 dB that corresponds to current attenuation of $p_{a}^{RF}$ = 3 dB and α = 18°.

Again, in the text we report only final equivalent values of the attenuator.

References

(1) Lumata, L.; Kovacs, Z.; Sherry, A. D.; Malloy, C.; Hill, S.; van Tol, J.; Yu, L.; Song, L.; Merritt, M. E. Electron Spin Resonance Studies of Trityl OX063 at a Concentration Optimal for DNP. *Phys. Chem. Chem. Phys.* **2013**, *15* (24), 9800. https://doi.org/10.1039/c3cp50186h.
